# New genus and two new species of Hyaliodini from the Philippines (Miridae, Deraeocorinae)

**DOI:** 10.3897/zookeys.796.21353

**Published:** 2018-11-15

**Authors:** Katrina L. Menard, Cameron D. Siler

**Affiliations:** 1 Sam Noble Oklahoma Museum of Natural History, University of Oklahoma, Norman, OK 73072-7029 University of Oklahoma Norman United States of America; 2 Department of Biology, University of Oklahoma, Norman, OK 73072-7029 University of Oklahoma Norman United States of America

**Keywords:** Bicol Peninsula, biodiversity, Caraballo Mountain Range, Luzon Island, northern Philippines

## Abstract

*Philicoris*, a new genus of the mirid subfamily Deraeocorinae, tribe Hyaliodini, is described from the Philippines. New species *Philicorismayon***sp. n.** and *Philicorispalali***sp. n.** from the island of Luzon are documented with photographic images of the dorsal habitus and male genital structures.

## Introduction

The island nation of the Philippines is home to a unique and highly endemic fauna, and as such is one of a small number of nations recognized globally as both a conservation biodiversity hotspot (Myers et al. 2000) and a megadiverse nation (Mittermeier et al. 1997). The capital city of Manila is on Luzon, a large island in the northern Philippines formed historically by the accretion of separate paleo-islands in the geological past (Adams and Pratt 1911; Rutland 1968; Hashimoto 1981a, b; Auffenberg 1988; Hall 1996; 1998; Yumul et al. 2009; Siler et al. 2011; Brown et al. 2013). It is assumed that the isolation and subsequent accretion of these precursor paleo-islands contributed to the evolutionary diversity found today (Brown et al. 1996, 2013; Brown and Diesmos 2009; Siler et al. 2011). However, our understanding of the impact Luzon’s complex geography and distinct subfaunal regions had on the diversification of terrestrial species continues to be hampered by limited knowledge of distribution patterns of the islands vertebrate and invertebrate faunas (Siler et al. 2011; Brown et al. 2013). A resurgence in studies of Luzon’s biodiversity, particularly for terrestrial vertebrates (reviewed by Brown and Diesmos 2009; Siler et al. 2011; Brown et al. 2013), has taken place over the last two decades. These studies have led to the discovery of several new species that appear restricted to volcanic peaks (i.e., Mts. Banahao, Isarog, Mayon; Heaney et al. 1999; Brown et al. 1995; Siler et al. 2017) or one of several distinct mountain ranges distributed across the island (i.e., Sierra Madres, Cordillera, and Caraballo Mountain ranges (Brown et al. 1996, 2012, 2013; Fuiten et al. 2011; Siler et al. 2009, 2013, 2014). Unfortunately, the diversity and distributions for members of many terrestrial organisms continue to be poorly understood throughout Luzon, including large gaps in our understanding of invertebrate diversity (Brown et al. 2013).

Diversity of Miridae in the Philippines remains unexplored for most of the major subfamilies and tribes. Within the eight currently recognized subfamilies and more than 11,130 species (Schuh 2013; Ferreira et al. 2015), only about 150 species are described from the Philippines (Schuh 2013). The island’s known diversity, however, explodes with a focused descriptive effort. One of the first works on Miridae in the Indo-Pacific, which included the Philippines, was that of Poppius (1915), who described 20 species from the island. The next intensive work on Philippine mirids is Schuh’s (1984) revision of the Indo-Pacific Phylinae, with 58 species recognized or described from the islands, including one endemic genus (*Abuyogocoris* Schuh). Almost all the material used in these descriptions was from general collecting expeditions in the Philippines in the 1960s; specimens are housed primarily in the Bishop Museum in Honolulu, Hawaii, and the American Museum of Natural History in New York (Schuh 1984). Since then, there has been little to no mirid-specific collecting on the islands, and few descriptions of new Philippine mirid taxa since the mid- to late 1980s (e.g., Schuh 1984; Stonedahl 1988).

**Figure 1.**
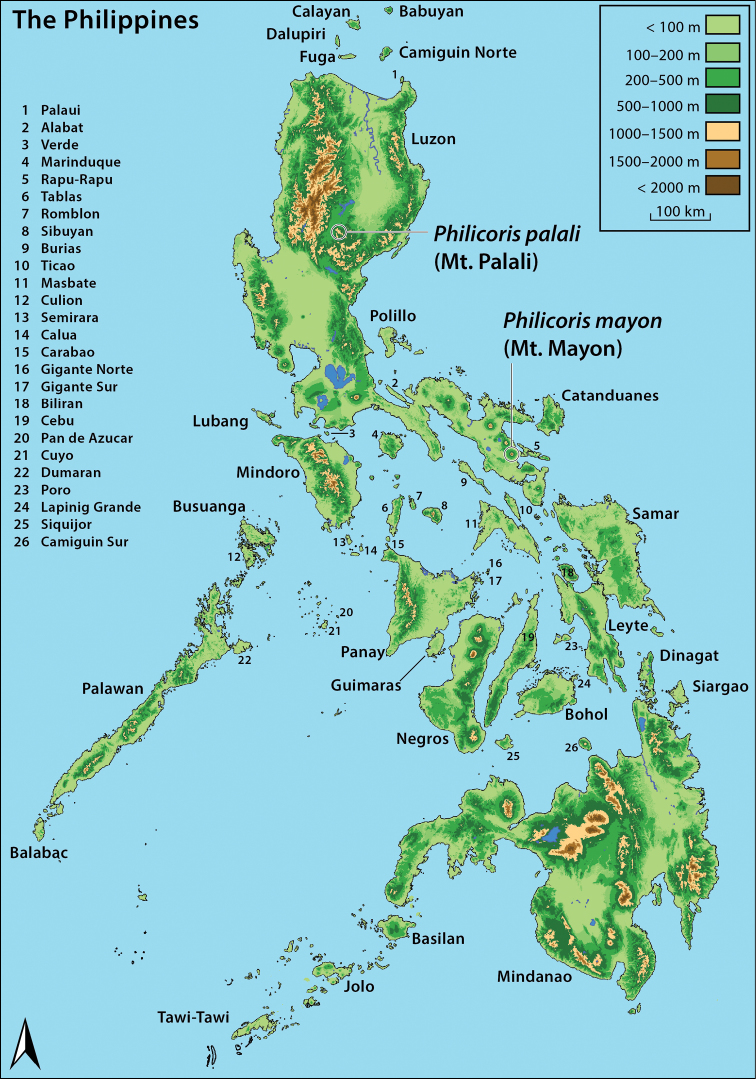
Topographic map of the Philippine archipelago, with island names provided for larger islands. Numeric labels for smaller islands correspond to inset key. Type localities on Luzon Island for *Philicorismayon* sp. n. (Mt. Mayon) and *Philicorispalali* sp. n. (Mt. Palali) shown for reference.

During faunal surveys in 2016 and 2017, specimens of two unique taxa of Miridae in the subfamily Deraeocorinae were captured among low-lying scrubs adjacent to palm farms, one on the foothill of the Mt. Mayon volcano of the southern Bicol Peninsula of Luzon Island, and the other at mid-elevation on Mt. Palali in the Caraballo Mountain Range of central Luzon Island (Fig. 1). The two taxa represent new species and together with a new genus, possess a suite of diagnostic morphological features that readily differentiate them from other mirid diversity. Both species possess the pretarsus with the basal tooth on the claw, the smooth and punctate dorsal surface of the thorax and hemelytron that are consistent with the subfamily (Ferreira et al. 2015). The Deraeocorinae comprise the tribes Clivinemini, Deraeocorini, Hyaliodini, Saturniomirini, Surinamellini, and Termatophylini. Specimens of *Philicoris* have both the hyaline membrane and the wide emboliar margin of the corium (Ferreira et al. 2015), which is consistent with the Hyaliodini Carvalho and Drake. The genus *Philicoris* does not possess the elongate anal tube in both males and females (Ferreira et al. 2015), or the stridulatory structures on the embolium (Akingbohungbe 1979), but these characters are not consistent across all genera of the tribe (e.g., *Linnavuorista* Akingbohungbe). No genera of Hyaliodini are recorded from the Philippines (Schuh 2013), and we are unable to key the newly collected specimens to any known genera in Akingbohungbe’s (1979) world key.

In this paper, external and internal genital features are used to demonstrate that both mirid populations on Luzon Island represent distinct evolutionary lineages (Wiley 1978; de Queiroz 1998, 1999) and are worthy of taxonomic recognition as members of a new genus in the tribe Hyaliodini. The recognition of these taxa represents the first records of the Hyaliodini in the Philippines. A new genus and two new species are described, technical images of key diagnostic traits provided, and its natural history, ecology, and geographic distribution are discussed. This manuscript was inspired by Dr. Thomas Henry, and this Festschrift article is dedicated to him. He never left a plant unsampled in fieldwork, and described hundreds of mirids across the world.

## Materials and methods

All specimens were collected by KLM in 2016 and 2017 as part of Sam Noble Oklahoma Museum of Natural History expeditions to the Philippines in conjunction with the National Science Foundation Grant (NSF IOS 1353683) to CDS. As part of the Memorandum of Agreement with the Philippines, all specimens are temporarily deposited at the Sam Noble Oklahoma Museum of Natural History, Norman, Oklahoma, United States (OMNH).

Stacked habitus photos were taken at the Sam Noble Museum Invertebrate Paleontology Stacking Photography Lab. Color photos and illustrations were edited using Adobe Photoshop CS4 and numbered in Adobe Illustrator CS4. Helicon Focus v4.2.9 software was used for all image stacking.

Scanning Electron Micrographs (SEM) were taken at the Samuel Roberts Noble Microscopy Laboratory at the University of Oklahoma. Dried specimens attached to paper points were removed from the pins, and the points used as mounts to attach to aluminum stubs with carbon conductive tape. The stubs and specimens were then coated with gold/palladium and examined with a Zeiss NEON 40 EsB SEM or a LEO 1450VP SEM.

Terminology for male genitalia follows Cassis (2008). Genitalia were extracted by soaking the abdomen in 85% lactic acid solution for one to a few days at room temperature, and then washing with distilled water before being dissected in glycerol. Images of the male genitalia were taken using an Olympus CX31 compound microscope with an Olympus 5MP CCD Camera using Cellsens Software. Helicon Focus v4.2.9 software was used for all image stacking.

Measurements were taken using an eyepiece micrometer (10mm/100×) on an Olympus SZX2 stereomicroscope following the methodology of Schuh (1984). Measurements include the body length (clypeus to cuneus), pronotum length (anterior margin to posterior margin), second antennal segment length, head width (distance between lateral margins of the eyes), pronotum width (width at widest point along the posterior margin), width at the widest point of the hemelytra, and interocular distance. All measurements are in millimeters.

## Taxonomy

### Philicoris gen. n.

Taxon classificationAnimaliaHemipteraMiridae

http://zoobank.org/FCC6FDF2-7C71-4F59-9C4E-9F78C26414CE

Figs 2 , 3 , 4 , 5

#### Type species.

*Philicorismayon* by original designation.

#### Included species.

*Philicorismayon* sp. n., *Philicorispalali* sp. n.

#### Diagnosis.

Recognized by trapezoidal and convexly rounded pronotum, shiny dorsum with punctation on pronotum and distinct rows of punctures along clavus and corium of hemelytron, wide emboliar margins, and shape of male genitalia.

#### Description.

***Male.* Coloration.***Head*: tan to light yellowish brown, clypeus and labrum dark brown; labium basally yellow and dark brown apically; first antennal segment dark brown, basally dark brown, transitioning to tan or orange to light brown distally with dark brown apices, second antennal segment contiguously tan or yellow transitioning to reddish brown then dark brown at distal apex, third antennal segment tan or white basally transitioning to dark brown distally, fourth antennal segment tan or dark brown. *Thorax*: collar tan or yellowish brown, pronotum tan or light yellowish brown, calli tan with posterior margin with lighter yellow macula or orange tinge along margin, mesoscutum brown with lateral yellow macula or light yellowish brown, scutellum dark brown medially and light whitish green along majority of lateral margins or light yellowish brown, lateral sclerites of thorax same coloration as pronotum or darker, scent gland and evaporative area same color as lateral sclerites or contrastingly whitish, procoxae light yellow or orange-brown, remaining coxae light brown apically and yellowish or orange-brown distally, femora tan or light yellowish brown with two orange-red stripes on preapical distal margins, and tibiae tan or orange and light yellowish brown basally, tarsomeres tan or light yellow. *Hemelytra*: embolium tan or light yellowish brown with orange tinge, cuneus tan or light yellowish brown with orange tinge, lateral margins of corium tan or light yellowish brown transitioning to light brown, clavus dark brown with light tan area along anterior one-third margin or with corium completely dark brown, membrane light brown to beige with orange-brown or beige veins. *Abdomen*: tan with dark brown anterior and posterior surfaces or completely-orange brown, gonopore tan or orange-brown. **Surface and vestiture**: *Head*: smooth and shiny, clothed with simple setae, antennal segments covered with dense simple setae of uniform length. *Thorax*: collar and calli smooth, remaining surface of pronotum punctate, covered with simple setae, scutellum and lateral sclerites of thorax with simple setae. *Hemelytra*: simple setae covering hemelytral surface, hemelytra surface shiny with dense punctuation in regular rows on clavus and corium, dorsal surface of emboliar margins and cuneus smooth. *Abdomen*: clothed with simple setae. **Structure**: *Head*: wider than high, clypeus not visible in dorsal view, frons convex, vertex flat, declining posteriorly towards anterior pronotal margin, eyes relatively large, taking up most of head in lateral view, dorsal surface confluent with vertex, posterior margin removed from anterior margin of pronotum, interocular width either greater than or less than width of single eye, first antennal segment length wider than interocular distance, less than half length of second segment, second antennal segment longest, apically narrower than first segment, distally widening to width equivalent to first, antennal segments three and four half width of antennal segment one, individually nearly equidistant in length to segment one; apex of labium extending to metacoxae. *Thorax*: pronotal collar narrow and rounded, dorsal surface of pronotum convexly rounded, anterior and posterior portions of pronotum not demarcated, pronotum trapezoidal with nearly straight lateral margins, calli fused into single weakly protruding plate surrounded by weakly defined rows of punctures, posterior margin of pronotum straight, mesoscutum mostly hidden or not visible, scutellum tumid, metathoracic scent gland relatively large, taking up greater than half area of metathoracic sclerite (Fig. 3A), femoral length equivalent and approximately four-fifth length of tibiae, metafemora greatest in width, medial width widest medially, tibial length nearly equivalent to emboliar length, third segment of pretarsus longest, pretarsal claws with basal tooth (Fig. 3B), parallel hair-like parempodia, lacking pulvilli. *Hemelytra*: weakly transversely rounded, lateral margins straight or weakly concave with wide embolium, cuneus longer than wide, bent ventrally at fracture, membrane with two visible veins forming two cells, larger cell length greater than one half total length of membrane. *Abdomen*: shorter than half total body length, relatively narrow, width tapering to gonopore. *Genitalia*: Endosoma primarily membranous with two spicules apically, preapical secondary gonopore and several membranous apical lobes (Figs 4A, 5A), phallotheca thin and simple, left paramere tall and crow-bar shaped, sometimes with basal spine projecting perpendicularly to base (Figs 4C, 5C), right paramere small, leaf-shaped, sometimes with apical bifurcation (Figs 4B, 5B).

**Figure 2.**
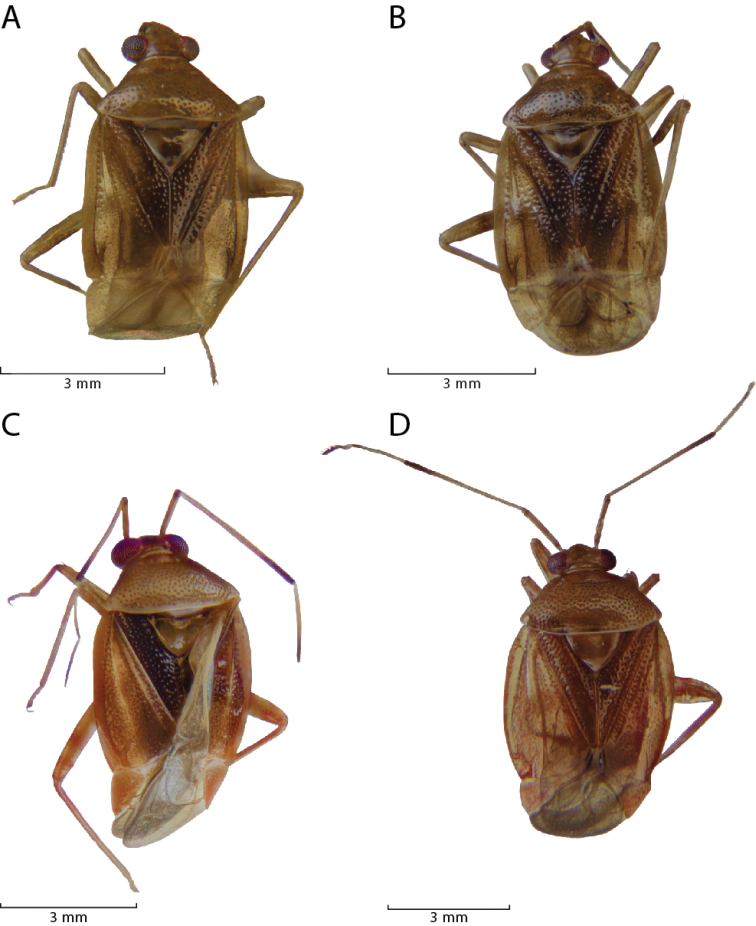
Dorsal habitus images of *Philicorismayon* sp. n. (**A** male OMNH 66500; **B** female OMNH 66501) and *Philicorispalali* sp. n. (**C** male OMNH 7804; **D** female OMNH 7805).

**Figure 3.**
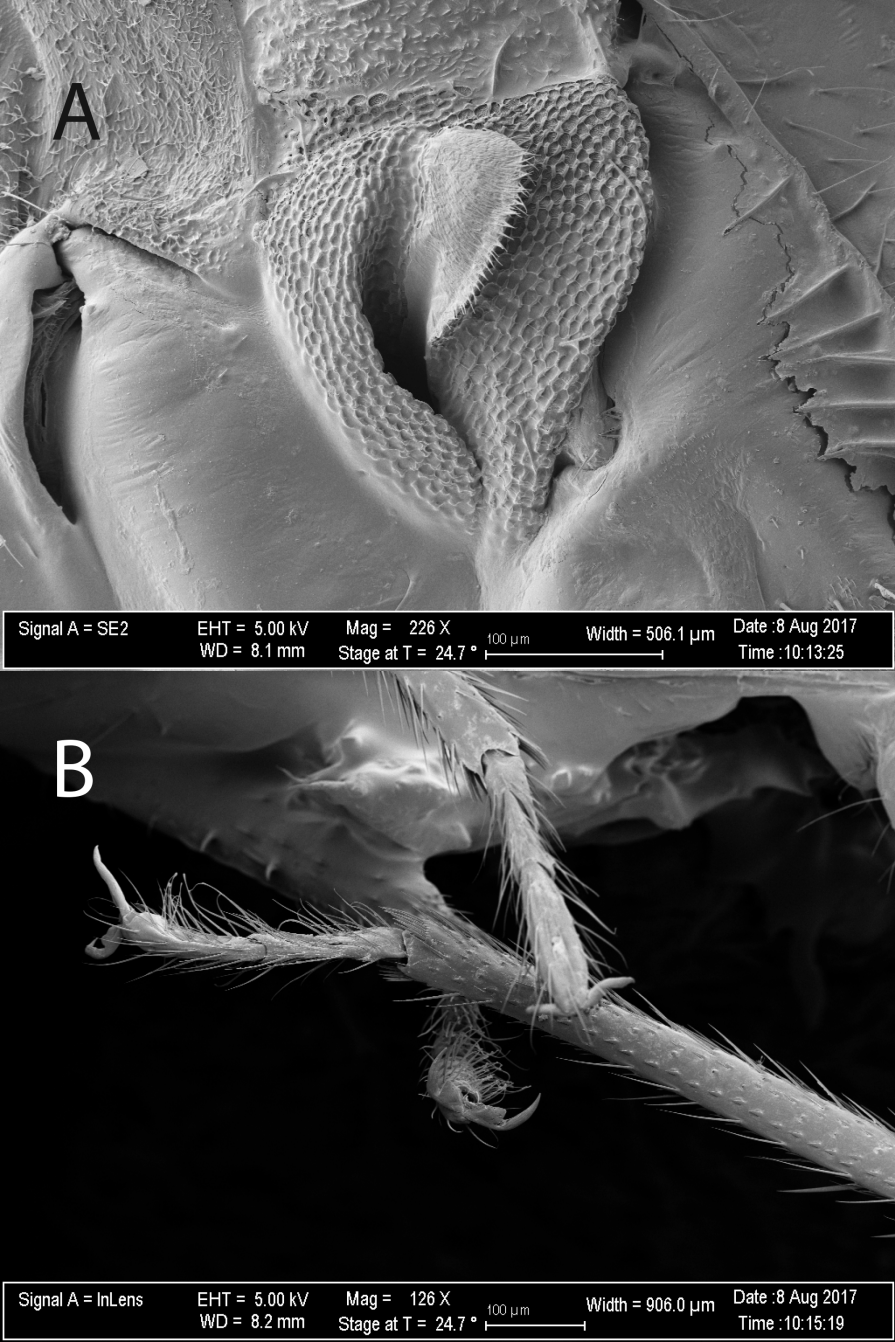
Scanning Electron Micrographs of *Philicorismayon* sp. n. visualizing the **A** scent gland evaporative area, and **B** the pretarsus.

**Figure 4.**
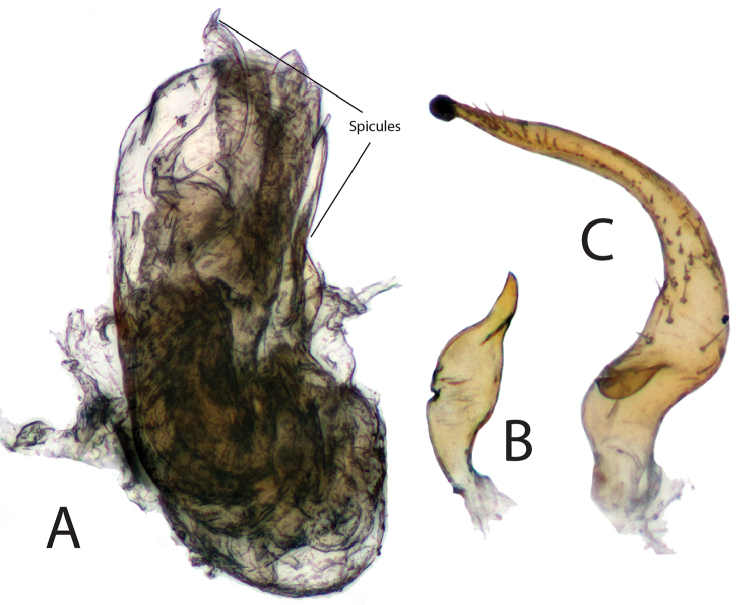
Male genitalia of *Philicorismayon* sp. n. showing **A** endosoma **B** right paramere, and **C** left paramere. Images taken at 100× using a compound light microscope.

**Figure 5.**
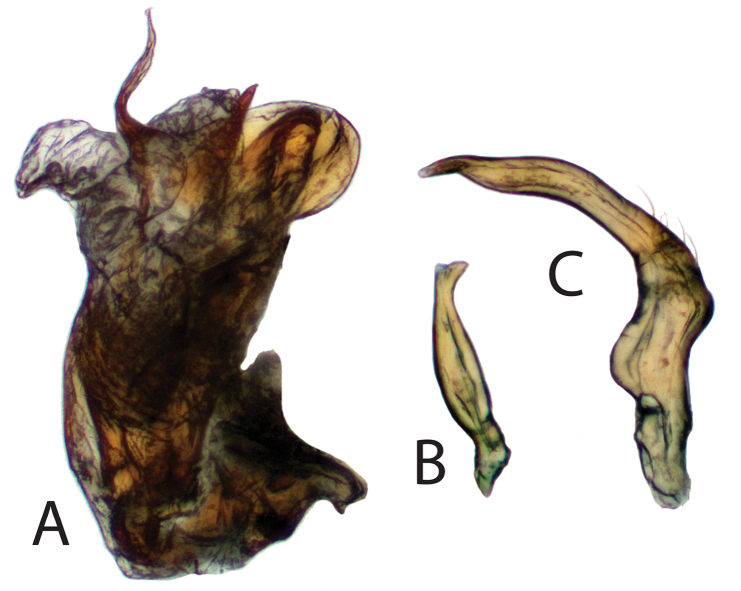
Male genitalia of *Philicorispalali* sp. n. showing **A** endosoma **B** right paramere, and **C** left paramere. Images taken at 100× using a compound light microscope.

***Female.*** Similar to males in coloration, surface and vestiture. Interocular distance greater in females, width of pronotum sometimes wider. *Genitalia*: not dissected.

#### Etymology.

The generic name is derived from the country of their discovery (Philippines) and “coris,” meaning bug in Latin. The gender is masculine.

#### Hosts.

Asteraceae.

#### Distribution.

Luzon Island, Philippines.

#### Discussion.

The combination of the basally toothed claw, the relatively wide embolium along the lateral margins of the hemelytron, the shiny and punctate pronotum and hemelytron, and the membranous endosoma clearly place this genus in the Hyaliodini as it is currently defined. Both species also have two apical endosomal spicules and multiple membranous lobes that correspond to other members of Deraeocorinae and Hyaliodini (Akingbohungbe 1979).

*Philicoris* is similar to the Neotropical genus *Antias* Distant in size and lacks a demarcation between the anterior and posterior portions of the pronotum but instead is tumid, and the hemelytron is not completely hyaline lines (Carvalho 1982). The calli are also not well developed. Unlike *Antias*, however, *Philicoris* has a smooth membrane without any setae, the eyes do not cover the entire area of the head in lateral view, and *Philicoris* has rows of punctation on the surface of the corium, embolium and claval suture lines. *Philicoris* also differs from the African genera *Obudua* Linnavuori and *Linnavuorista* Akingbohungbe by the lack of a narrowed anterior margin, the former by the lack of distinctly swollen calli, and from the latter by lacking a distinct apical spur on the metafemur. The Palearctic and African genus *Stethoconus* Flor also differs from *Philicoris*; the latter lacks an anterior constriction to the pronotum, has a narrow collar, and has a relatively concolorous pronotum and hemelytron versus the highly patterned *Stethoconus*. For these reasons, we argue that this taxon represents a new genus.

### Philicoris mayon sp. n.

Taxon classificationAnimaliaHemipteraMiridae

http://zoobank.org/C830548D-70EE-4749-902B-CC8F3D0DCA92

Figs 2 , 3 , 4

#### Holotype.

Adult male (OMNH 7804) collected by K. Menard during daytime surveys on 04 March 2016, on the foothills of Mt. Mayon, Sitio Nagsipit, Barangay Mariroc, Municipality of Tabaco, Albay Province, Luzon Island, Philippines (13.3056'N, 123.687'E; datum = WGS84; 399 m elevation).

#### Paratypes.

One adult male (OMNH 7803) and one female (OMNH 7805), collected during daytime surveys on 11 March 2016, on the foothills of Mt. Mayon, Sitio Nagsipit, Barangay Mariroc, Municipality of Tabaco, Albay Province, Luzon Island, Philippines (13.30563'N, 123.6896'E; datum = WGS84; 382 m elevation).

#### Diagnosis.

Distinguished from its congener by the following combination of characters: (1) reddish orange coloration on lateral margins of hemelytron and legs; (2) extensive and defined rows of punctuation; (3) interocular distance relatively narrow; and (4) endosomal spicules weakly sclerotized.

#### Description.

***Male.* Coloration.***Head*: light yellowish brown, clypeus and labrum dark brown; labium yellow basally and dark brown apically; first antennal segment light orange-brown with dark brown apices, second antennal segment yellow transitioning to reddish brown then dark brown at distal apex, third antennal segment white basally, dark brown distally, fourth segment completely dark brown. *Thorax*: light yellowish brown, punctures orange-brown, collar lighter yellow, margins of calli more orange, mesoscutellum and scutellum light yellowish brown as pronotum, lateral sclerites of thorax same coloration as pronotum or darker brown, scent gland with same coloration as lateral sclerites, coxae and trochanters light orange-brown, femora light yellowish brown with two orange-red stripes on preapical distal margins, tibiae orange basally and light yellowish brown distally, tarsomeres light yellow. *Hemelytra*: light yellowish brown with orange tinge, orange tinge most predominant along emboliar margins and cuneus, clavus darker orange-brown to dark brown, corium darker yellowish brown, membrane pale brown with orange veins. *Abdomen*: orange-brown. **Surface and vestiture**: *Head*: smooth and shiny, clothed with dense simple setae, antennal segments covered with simple setae of uniform length. *Thorax*: collar and calli smooth, remaining surface of pronotum punctate, covered with dense simple setae, scutellum and lateral sclerites of thorax with simple setae. *Hemelytra*: simple setae covering hemelytral surface, hemelytra surface shiny with dense punctuation in regular rows on clavus, and corium, dorsal surface of emboliar margins and cuneus smooth. *Abdomen*: clothed with simple setae. **Structure**: *Head*: wider than high, clypeus not visible in dorsal view, frons convex, vertex flat, declining posteriorly towards anterior pronotal margin, eyes relatively large, taking up most of head in lateral view, dorsal surface confluent with vertex, posterior margin removed from anterior margin of pronotum, interocular width narrower than width of single eye, first antennal segment length wider than interocular distance, less than half length of second segment, second antennal segment longest, apically narrower than first segment, distally widening to width equivalent to first, antennal segments three and four half width of antennal segment one, individually nearly equidistant in length to segment one; apex of labium extending to metacoxae. *Thorax*: pronotal collar narrow and rounded, dorsal surface of pronotum convexly rounded, anterior and posterior portions of pronotum not demarcated, pronotum trapezoidal with nearly straight lateral margins, calli fused into single weakly protruding plate surrounded by well-defined rows of punctures, posterior margin of pronotum straight, mesoscutum mostly hidden or not visible, scutellum tumid, metathoracic scent gland relatively large, taking up greater than half area of metathoracic sclerite (Fig. 3A), pretarsal claws with basal tooth, parallel hair-like parempodia, pulvilli absent (Fig. 3B). *Hemelytra*: weakly transversely rounded, lateral margins convex with relatively wide embolium, cuneus longer than wide, bent ventrally at fracture, membrane with two visible veins forming two cells, larger cell length greater than one half total length of membrane. *Abdomen*: shorter than half total body length, relatively narrow, width tapering to gonophore. *Genitalia*: Endosoma primarily membranous with two weakly sclerotized spicules and preapical secondary gonopore surrounded by several membranous apical lobes (Fig. 4A), phallotheca thin and simple, left paramere tall and scythe-shaped with basal spine projecting perpendicularly to base and apex with round “hook” (Fig. 4C), right paramere small, leaf-shaped with apex pointed (Fig. 4B).

***Female.*** Similar to males in coloration, surface and vestiture. Pronotum wider in females, interocular distance greater. Genitalia not dissected.

#### Measurements (in mm for male/female).

male/female: tylus-cuneus length 2.50–2.70/2.80, hemelytron width 2.0–2.20/2.10, head width 0.85–0.90/0.85, interocular distance 0.28–0.30/0.35, pronotum length 0.75–0.80/0.75, pronotum width 1.50–1.55/1.75, antennal segment I length 0.60/0.65, antennal segment II length 1.50–1.55/1.55.

#### Etymology.

Named for Mt. Mayon, the type locality. Noun in apposition.

#### Hosts.

Unknown.

#### Distribution.

Luzon Island, Philippines.

#### Discussion.

This species was found on an unidentified prostrate plant in a relatively agricultural area of Mt. Mayon, around banana and palm farms. Its coloration is unique and roughly matches the pinkish red flowers of the plant it was found on.

### Philicoris palali sp. n.

Taxon classificationAnimaliaHemipteraMiridae

http://zoobank.org/ECD59B10-F399-4261-8383-BDBAE63A6741

Figs 2 , 5

#### Holotype.

Adult male (OMNH 65500), hand collected by K. Menard during daytime surveys on 09 June 2017 on the foothills of Mt. Palali, Municipality of Quezon, Nueva Vizcaya Province, Luzon Island, Philippines (16.45985'N, 121.22316'E; datum = WGS84).

#### Paratype.

One adult female (OMNH 65501), same information as holotype.

#### Diagnosis.

Recognized by mostly tan overall coloration, lateral pale greenish maculation on scutellum, white scent gland, tan thorax, interocular distance wider than width of eye, relatively narrower and straight emboliar margin, less prominent and defined surface punctuation, left paramere lacking basal perpendicular spine, and right paramere apically bifurcate.

#### Description.

***Male.* Coloration.***Head*: tan, clypeus and labrum dark brown; labium basally yellow and dark brown apically; first antennal segment basally dark brown, distally transitioning to tan, remaining segments tan. *Thorax*: collar and pronotum tan, calli tan with posterior margin with lighter yellow macula, mesoscutum brown with lateral yellow macula when visible, scutellum dark brown medially and light whitish green along majority of lateral margins, apex yellow, lateral sclerites of thorax same coloration as anterior pronotum, scent gland and evaporative area whitish, procoxae light yellow, remaining coxae light brown apically, yellowish distally, femora and tibiae tan, tarsomeres tan. *Hemelytra*: embolium and cuneus tan, lateral margins of corium tan transitioning to light brown, clavus dark brown with light tan area along anterior 1/3 of margin with corium, and corium, membrane and veins beige. *Abdomen*: tan with dark brown anterior and posterior surfaces, gonopore tan. **Surface and vestiture**: *Head*: smooth and shiny, clothed with simple setae, antennal segments covered with dense simple setae of uniform length. *Thorax*: collar and calli smooth, remaining surface of pronotum punctate, covered with simple setae, scutellum and lateral sclerites of thorax with simple setae. *Hemelytra*: surface with simple setae, shiny, with dense punctuation in regular rows on clavus and corium, dorsal surface of emboliar margins and cuneus smooth. *Abdomen*: clothed with simple setae. **Structure**: *Head*: wider than high, clypeus not visible in dorsal view, frons convex, vertex flat, declining posteriorly towards anterior pronotal margin, eyes relatively large, taking up most of head in lateral view, dorsal surface confluent with vertex, posterior margin removed from anterior margin of pronotum, interocular width greater than width of single eye, first antennal segment length wider than interocular distance, less than half length of second segment, second antennal segment longest, apically narrower than first segment, widening distally to width equivalent to first, antennal segments three and four half width of antennal segment one, individually nearly equidistant in length to segment one; apex of labium extending to metacoxae. *Thorax*: pronotal collar narrow and rounded, dorsal surface of pronotum convexly rounded, anterior and posterior portions of pronotum not demarcated, pronotum trapezoidal with nearly straight lateral margins, calli fused into single weakly protruding plate surrounded by weakly defined rows of punctures, posterior margin of pronotum straight. *Hemelytra*: weakly transversely rounded, lateral margins straight with relatively wide embolium, cuneus longer than wide, bent ventrally at fracture, membrane with two visible veins forming two cells, larger cell length greater than one half total length of membrane. *Abdomen*: shorter than half total body length, relatively narrow, width tapering to gonophore. *Genitalia*: endosoma primarily membranous with two sclerotized spicules surrounded by several membranous apical lobes (Fig. 5A), phallotheca thin and simple, left paramere scythe-shaped without spine projecting perpendicularly to base (Fig. 5C), right paramere small, leaf-shaped with apical bifurcation (Fig. 5B).

***Female.*** Similar to males in coloration, surface and vestiture. Interocular distance greater in females. Genitalia not dissected.

#### Measurements

**(in mm for male/female).** Tylus-cuneus length 2.10/2.40, hemelytron width 1.55/1.70, head width 0.75/0.70, interocular distance 0.30/0.38, pronotum length 0.75/0.75, pronotum width 1.40/1.45, antennal segment I length 0.45/0.50, antennal segment II length 1.40/1.35.

#### Etymology.

We name the new species in reference to Mt. Palali, the type locality. Noun in apposition.

#### Hosts.

Purple composite (Asteraceae).

#### Distribution.

The new species is known from mid-elevation habitats at the type locality on Mt. Palali, Nueva Vizcaya Province, Luzon Island, Philippines.

#### Discussion.

This species was found by sweeping an unidentified purple composite along a trail up Mt. Palali, approximately 800 m away from an area cleared for banana and agricultural cultivation. Therefore, the host plant might be an introduced species in the regional flora. The additional sampling of local plants, including varieties introduced for agriculture, may yet yield additional new species.

## Supplementary Material

XML Treatment for Philicoris

XML Treatment for Philicoris mayon

XML Treatment for Philicoris palali
